# Multiple-testing correction in metabolome-wide association studies

**DOI:** 10.1186/s12859-021-03975-2

**Published:** 2021-02-12

**Authors:** Alina Peluso, Robert Glen, Timothy M. D. Ebbels

**Affiliations:** 1 Division of Systems Medicine, Department of Metabolism, Digestion and Reproduction, Imperial College London, South Kensington Campus, London, SW7 2AZ UK; 2 Department of Chemistry, Centre for Molecular Informatics, University of Cambridge, Cambridge, CB2 1EW UK

**Keywords:** FWER, MWAS, MWSL, Multiple testing, Permutation, Correlated tests

## Abstract

**Background:**

The search for statistically significant relationships between molecular markers and outcomes is challenging when dealing with high-dimensional, noisy and collinear multivariate omics data, such as metabolomic profiles. Permutation procedures allow for the estimation of adjusted significance levels without assuming independence among metabolomic variables. Nevertheless, the complex non-normal structure of metabolic profiles and outcomes may bias the permutation results leading to overly conservative threshold estimates i.e. lower than those from a Bonferroni or Sidak correction.

**Methods:**

Within a univariate permutation procedure we employ parametric simulation methods based on the multivariate (log-)Normal distribution to obtain adjusted significance levels which are consistent across different outcomes while effectively controlling the type I error rate. Next, we derive an alternative closed-form expression for the estimation of the number of non-redundant metabolic variates based on the spectral decomposition of their correlation matrix. The performance of the method is tested for different model parametrizations and across a wide range of correlation levels of the variates using synthetic and real data sets.

**Results:**

Both the permutation-based formulation and the more practical closed form expression are found to give an effective indication of the number of independent metabolic effects exhibited by the system, while guaranteeing that the derived adjusted threshold is stable across outcome measures with diverse properties.

## Background

In omics studies many hundreds to tens of thousands of molecular variables are collected for each individual, leading to high-dimensional multivariate data which are highly collinear. When analysing these data, many hypothesis tests are conducted simultaneously, thus effective methods to adjust for multiple testing are a central topic, especially in the context of Metabolome-Wide Association Studies (MWAS) [1]. The aim is the detection of statistically significant relationships between molecular concentrations and disease outcomes while minimising the risk of false positive associations. A widely used approach for multiple testing is the false discovery rate (FDR) [2] which controls the expected proportion of falsely rejected hypotheses among all those rejected. This approach is effective in the case of independent or positive dependent tests. While there have been some attempts to deal with correlated tests such as [3] that proposed a simple but highly conservative procedure, in general correlation among tests is still a problem for FDR methods. Besides FDR corrections, family-wise error rate (FWER) procedures control the probability of making at least one false conclusion (i.e. at least one Type I error). The FWER provides a more stringent control of Type I error compared to the FDR. Nevertheless, conventional FWER methods such as the Bonferroni [4] or Sidak [5] adjustment are known to be overly conservative when the tests are correlated. On the other hand, resampling-based methods such as the permutation test are a standard tool to simultaneously assess the association of different correlated molecular quantities with an outcome of interest. These procedures can be conducted in both a parametric or non-parametric fashion. Parametric approaches are the preferred methods as they have relatively high power if the assumptions (e.g. normality of the data) hold. Nevertheless, in the context of MWAS the metabolic profiles are very rarely normally distributed nor present a symmetric distribution, and this may bias the result of the chosen significance test.

Thus, a first aim of this study is to overcome this issue and derive a valid yet stable metabolome-wide significance level (MWSL) across outcomes with diverse distributional properties. The proposed approach is based on a permutation procedure built from parametric approximation methods via the multivariate Normal and log-Normal distributions to describe the set of metabolic profiles while retaining their complex correlated structure up to the 2nd order moments, while effectively controlling the expected overall type I error rate at the $\alpha$ level. While the proposed re-sampling-based method is accurate and asymptotically consistent it demands intensive computation. In the context of genomic studies there have been several attempts to formulate the problem in terms of estimating the number of non-redundant molecular quantities as a closed-form eigenvalue-based measure from the spectral decomposition of the empirical correlation matrix of the molecular variables. The available measures proposed by [6–9], and [10] are found not to be sufficiently accurate as a valid substitute for the proposed permutation procedure. Therefore, a second aim of this study is to derive a permutation-free closed-form estimate of the MWSL to express the number of non-redundant molecular quantities. Both the permutation-based MWSL formulation and the more practical closed form expression are tested on synthetic and real data.

## Methods

### Permutation-based MWSL estimation

#### Permutation algorithm

Suppose the data consists of *n* observations, and let *Y* be the outcome of interest, $X=(X_{1},\ldots ,X_{M})^T$ the vector of *M* predictors or features, and $Z=(Z_{1},\ldots ,Z_{P})^T$ the vector of *P* fixed effect covariates. The permutation-based MWSL estimation can be described as follows.

- Step (1): Shuffle i.e. re-sample without replacement, the outcome variable *Y* together with the set of fixed effects confounders *Z* if any. In this way, the *n* subjects are re-sampled under the null hypothesis of no association.
- Step (2): To estimate the relationship between the outcome and the set of features while accounting for possible confounding effects, compute *M* regression models in a univariate approach, that is by using one feature at a time. From each model store the *p* value associated with the feature of interest. When appropriate, approaches other than regression methods can be used for testing of association e.g. correlation or t-test.
- Step (3): Extract the minimum of the set of *M* *p* values as this indicates the highest threshold value which would reject all *M* null hypotheses.
- Step (4): Repeat Step (1)–(3) for *K* times, where *K* is at least *n*/2 times [11]. The *K* minimum *p* values are the elements of the new vector *q*.
- Step (5): Sort the elements of *q*, and take the ($\alpha K$)-value of this vector. This value is the MWSL estimate. An approximate confidence interval can be obtained by treating the true position of the MWSL estimate as a Binomial random variable with parameters *K* and $\alpha$. Then, using the Normal approximation to the Binomial, we obtain the $z_{(1-\alpha )\%}$ confidence limits by extracting the elements of *q* in positions $(\alpha K)\pm \{ (1-\alpha ) \sqrt{\alpha K (1-\alpha )} \}$.
- Step (6): Compute the effective number of tests (ENT) defined as the number of independent tests that would be required to obtain the same significance level using the Bonferroni correction ENT = $\frac{\alpha }{\text {MWSL}}$. The ENT estimate measures the extent that the *M* markers are non-redundant. Therefore, the ratio R = $\frac{\text {ENT}}{M}\%$ of the effective and the actual number of tests (ANT or *M*) is a measure of the dependence among features, which is expected to be closer to 0% when highly correlated features are considered.

Permutation-based procedures have previously been applied in different studies e.g. by [12] to approximate the genome-wide significance threshold for dense SNP and resequencing data, or by [13] for urinary metabolic profiles. Recently in the context of NMR metabolic profiling studies [14] employed the permutation algorithm to perform a series of MWAS for serum levels of glucose. Counterintuitively, ENT estimates greater than the ANT were found, with an R ratio for glucose over 400%. With the methodology proposed in this paper, we generalise the algorithm to a more flexible regression context compared to [13], while we provide a robust framework to avoid biased estimates as in [14].

#### Parametric simulation methods

The underlying assumption of the permutation procedure is that the *p* values are properly calibrated, that is, every metabolite-specific *p* value is uniformly distributed, i.e. *p* value$_m$ $\sim U(0,1)$ where $m=1,\ldots ,M$, when the null hypothesis is true. Because the MWSL is the minimum *p* value across the metabolite specific tests, all it takes is one poorly calibrated test with an erroneous small *p* value to bias the MWSL estimation. Very often in metabolomics studies the features are not normally distributed. Nevertheless, normality only matters sometimes. It matters when both the feature and the outcome have a skew distribution [15], while it has very little effect when either the feature or the outcome is normally distributed. In this context, we investigate the properties of the permutation approach for significance level estimation by employing the multivariate Normal and the multivariate log-Normal distributions to describe, at least approximately, the set of correlated features and to obtain stable estimates of the MWSL while effectively controlling the maximum overall type I error rate at the $\alpha$ level. We assume that the data are already centred so that the means equal zero. Therefore, $X \sim {{\mathcal {N}}}_{M}(\mu , \Sigma ^*)$ is the multivariate Normal distribution employed to simulate the set of features where $\mu = \text {E}[X]=(\text {E}[X_{1}],\ldots ,\text {E}[X_{M}])^T={\mathbf{0 }}$ is the *M*-dimensional mean vector of zero means, and $\Sigma ^*$ is the $(M \times M)$ shrinkage estimator of the covariance matrix as described by [16]. The shrinkage estimator is always positive definite, well-conditioned, more efficient and therefore preferred to the unbiased estimator $\Sigma$, or to the related maximum likelihood estimator $\Sigma _{\text {ML}}$. Where the probability density of a feature is right skewed, we use the multivariate log-Normal approximation. In such cases, the features are first transformed i.e. the absolute value of their minimum, plus one unit, is added to their original value. The algorithm is applied to real-data and simulated scenarios to illustrate the results for different model parametrizations and various type of outcome, as well as to investigate different correlation levels across features and between the features and the outcome.

### Practical approximation of the ENT

The empirical method of computing the permutation test *p* value is hampered by the fact that a very large number of permutations are required to correctly estimate small, and therefore interesting *p* values. Thus, we now present a more efficient alternative to a standard permutation test to derive the MWSL. To distinguish from the effective number of non-redundant variates from the permutation procedure which has been defined as ENT in Section “Methods”, here we refer to the estimate from this practical approximation approach as Meff. It has previously been shown that the collective correlation among a set of variables can be measured by the variance of the eigenvalues ($\lambda$s) derived from a correlation matrix [6, 17]. In particular, high correlation among variables leads to high $\lambda$s, that is, when all variables are completely correlated, the first $\lambda$ equals the number of variables in the correlation matrix (M) and the rest of the $\lambda$s are zero. Vice versa in the case of no correlation among variables, all the $\lambda$s will be equal to 1 with zero variance. Hence, the variance of the $\lambda$s will range between zero, and *M*. Based on this concept, within the genomics field several methods have been proposed for estimating the ENT from the correlations between variates. Among the first, [7] proposed to use the variance of the $\lambda$s to estimate the ENT for the limiting cases of none/ fully correlated variables, and a ratio of the eigenvalue’s variance to its maximum *M* for intermediate situations. [8] suggested summing the $\lambda$s, after substituting 1 for the $\lambda$s that are greater than 1. [9] suggested defining ENT as the number of $\lambda$s which can explain a certain percentage of the variation within the data. However, it is unclear how the percentage should be chosen as an excessively large or small value would result in an FWER that is overly conservative or liberal. [10] proposed a measure of ENT based on a $\lambda$s ratio function. In the context of our analyses, the Meff measures proposed by these authors were not sufficiently accurate as a valid substitute for the permutation procedure, hence we propose an empirical closed-form expression directly related to the correlation among metabolomics variates as follows

$$\begin{aligned} \text {Meff}_{\text {MWSL}} = {\left( \frac{\sum _{m=1}^M{\sqrt{\lambda _m}}}{\log (\lambda _1)} \right) }^{2} \, \Big / \, \left( \frac{\sum _{m=1}^M{\lambda _m}}{\lambda _1} + \sqrt{\lambda _1} \right) . \end{aligned}$$

This formulation balances the information from the $\lambda _m$ with $m=1,\ldots ,M$ estimated eigenvalues from the correlation matrix of the metabolite concentrations, with the contribution of the first eigenvalue $\lambda _1$ which measures the primary cluster in the matrix, its number of variables, and the average correlation among the features [18]. This formulation is of interest in the context of correlated variates, that is when at least two variates are dependent, i.e. for $\lambda _1 >1$, and therefore $\log (\lambda _1) > 0$.

Next, the MWSL can be derived based on the following.

- Step (1): Compute the $\text {Meff}_{\text {MWSL}}$ with the proposed formulation.
- Step (2): The MWSL can be derived based on a Bonferroni correction i.e. MWSL = $\frac{\alpha }{\text {Meff}_{\text {MWSL}}}$ .

Alternatively, the full algorithm as an alternative to the permutation procedure can be described as follows.

- Step (1): Compute the $\text {Meff}_{\text {MWSL}}$ with the proposed formulation.
- Step (2): Under the null hypothesis the *p* value of each metabolite follows a Uniform distribution, i.e. *p* value$_m$ $\sim U(0,1)$, where $m=1,\ldots ,M$. The distribution of minimum *p* values can be approximated by the minimum order statistics (r=1), that is $U_{(1)} \sim$ Beta(1, *M*) in the case of not correlated molecular variates, and Beta$(1,M')$ with $M' \leqslant M$ in the case of correlated features. The limit case of very highly correlated features with $M'=1 (<<M)$ reduces to sampling from a Beta(1, 1) which equals a *U*(0, 1). It follows that the $\text {Meff}_{\text {MWSL}}$ can be used to approximate the distribution of minimum *p* values by sampling from a Beta$(1,\text {Meff}_{\text {MWSL}})$.
- Step (3): The MWSL and its respective $z_{(1-\alpha )}$% confidence limits can be derived as described in Section “Permutation algorithm”, Step (5)–(6) of the permutation procedure.

## Results

### Study of experimental metabolomics data

The MWAS approach was employed to investigate the association between human serum $^{1}$H NMR metabolic profiles and various clinical outcomes in the Multi-Ethnic Study of Atherosclerosis (MESA) [19]. The data have been extensively described in [14]. Briefly, the cohort includes participants (51% females, 49% males), aged 44-84 years, (mean=63 years) from four different ethnic groups: Chinese-American, African-American, Hispanic, and Caucasian, all recruited between 2000 and 2002 at clinical centres in the United States and free of symptomatic cardiovascular disease at baseline. Demographic, medical history, anthropometric, and lifestyle data, as well as serum samples were collected, together with information on diabetes, and lipid and blood pressure treatment. Metabolic profiles were obtained using $^{1}$H NMR at 600 MHz and processed as detailed in [20]. The outcomes of interest are glucose concentrations and the body mass index (BMI). Table 1 presents the descriptive statistics for the clinical outcome measures, while Table 2 reports the descriptive statistics for the fixed effects covariates used in the study. Three sets of NMR spectra are considered: (1) a standard water-suppressed one-dimensional spectrum (NOESY), (2) a Carr-Purcell-Meiboom-Gill spectrum (CPMG), and (3) a lower resolution version of the CPMG data (BINNED^1^). The BINNED version consists of *M*=655 features, while the NOESY and CPMG contain *M*=30,590 features. The BINNED data sample comprises of *n*=3,500 individuals, while the NOESY and CPMG data have *n*=3,867 individuals. All MWSL calculations are performed for $\alpha =0.05$.

**Table 1:** Descriptive statistics for the clinical outcome measures

Outcome	Mean	sd	Median	Min	Max	Skewness	Kurtosis
Glucose (mg/dL)	98.28	31.10	90	38	507	4.17	28.89
Logarithm of Glucose	4.56	0.23	4.5	3.64	6.23	2.22	10.35
BMI (kg/m$^2$)	28.14	5.39	27.34	15.36	61.86	46.50	4.45
Logarithm of BMI	3.32	0.18	3.31	2.73	4.12	1.39	3.20

**Table 2:** Descriptive statistics for the fixed effects covariates

Covariate	Mean	sd
Age (years)	62.89	10.32
Gender	0.51	0.49
Height (cm)	166.43	10.23
Ethnicity: Caucasian	0.39	0.49
Ethnicity: Hispanic	0.23	0.42
Ethnicity: African-American	0.25	0.43
Ethnicity: Chinese-American	0.13	0.34
Smoking: Never	0.51	0.50
Smoking: Former	0.12	0.33
Smoking: Current	0.38	0.48
LDL cholesterol (mg/dL)	117.67	31.04
HDL cholesterol (mg/dL)	51.29	14.41
Systolic blood pressure (mmHg)	126.92	21.54
Blood pressure treatment	0.38	0.49
Diabetes	0.14	0.34
Lipids treatment	0.17	0.37

**Table 3:** Real data: Comparison of estimation of the number of non-redundant variates from the permutation method (ENT, obtained as the average of the ENT estimates for all the clinical outcomes measures considered via the multivariate Normal and the multivariate log-Normal methods) and via approximation procedures based on the eigenvalues of the correlation matrix of the metabolite concentrations (Meff)

	MESA BINNED	MESA NOESY	MESA CPMG
ENT	352(53.8%)	2744(9.0%)	16014(52.3%)
Meff.MWSL	**345(52.7%)**	1931(6.3%)	**11570(37.8%)**
Meff.Galwey [10]	201(30.7%)	524(1.7%)	1815(5.9%)
Meff.Gao [9]	435(66.4%)	**2705(8.8%)**	3537(11.6%)
Meff.Liji [8]	226(34.5%)	2534(8.3%)	4972(16.3%)
Meff.Nyholt [7]	611(93.3%)	26823(87.7%)	29704(97.1%)

R = ENT/ANT(%) ratio in brackets. ANT = 655 for the MESA BINNED data, and ANT = 30590 for the NOESY and CPMG data. Meff estimates closest to ENT estimates in bold

**Table 4:** Real data: MWSL estimation comparison between the permutation method and the approximation procedure generating the distribution of the minimum *p* value as a Beta(1,Meff_MWSL_)

MESA BINNED			MESA NOESY			MESA CPMG		
	Beta(1,345)	Permutation		Beta(1,1931)	Permutation		Beta(1,11570)	Permutation
ENT/ANT(%)	**51.4%**	**53.8%**	ENT/ANT(%)	**6.2%**	**9.0%**	ENT/ANT(%)	**36.9%**	**52.3%**
ENT	336	352	ENT	1883	2744	ENT	11279	16014
ENT_CI.low	335	338	ENT_CI.low	1874	2439	ENT_CI.low	11229	14509
ENT_CI.up	338	369	ENT_CI.up	1891	3080	ENT_CI.up	11330	17208
MWSL	**0**.**0001486**	**0**.**0001410**	MWSL	**0**.**0000266**	**0**.**0000179**	MWSL	**0**.**0000044**	**0**.**0000031**
MWSL_CI.up	0.0001493	0.0001476	MWSL_CI.up	0.0000267	0.0000195	MWSL_CI.up	0.0000045	0.0000036
MWSL_CI.low	0.0001480	0.0001340	MWSL_CI.low	0.0000264	0.0000160	MWSL_CI.low	0.0000044	0.0000028

**Table 5:** Simulated data: Comparison of estimation of the number of non-redundant variates from the permutation method (ENT, obtained as the average of the ENT estimates for all the simulated uncorrelated and correlated outcomes measures considered via the multivariate Normal and the multivariate log-Normal methods) and via the approximation procedure based on the eigenvalues of the correlation matrix of the metabolite concentrations (Meff)

Correlation $\in$	[0.95-1)	[0.85,0.95)	[0.75,0.85)	[0.65,0.75)	[0.55,0.65)	[0.45,0.55)	[0.35,0.45)	[0.25,0.35)	(0,0.25)
ENT	3(0.5%)	11(1.7%)	36(5.4%)	81(12.4%)	134(20.5%)	210(32%)	319(48.7%)	433(66.1%)	554(84.6%)
Meff.MWSL	**4(0.6%)**	**14(2.2%)**	**40(6.2%)**	**74(11.4%)**	**126(19.4%)**	195(30%)	**282(43.4%)**	**416(64%)**	416(64%)
Meff.Nyholt	34(5.2%)	121(18.6%)	239(36.8%)	331(50.9%)	419(64.5%)	491(75.5%)	543(83.5%)	588(90.5%)	**638(98.2%)**
Meff.Liji	18(2.8%)	64(9.8%)	133(20.5%)	195(30%)	263(40.5%)	320(49.2%)	365(56.2%)	413(63.5%)	382(58.8%)
Meff.Gao	65(10%)	131(20.2%)	213(32.8%)	275(42.3%)	327(50.3%)	372(57.2%)	412(63.4%)	449(69.1%)	317(48.8%)
Meff.Galwey	7(1.1%)	23(3.5%)	58(8.9%)	98(15.1%)	147(22.6%)	**200(30.8%)**	252(38.8%)	312(48%)	270(41.5%)

R = ENT/ANT(%) ratio in brackets. ANT = 650. Meff estimates closest to ENT estimates in bold

**Table 6:** Simulated data: MWSL estimation comparison between the permutation method and the approximation procedure generating the distribution of the minimum *p* value as a Beta(1, Meff_MWSL_)

Correlation $\in$ **[0.95-1)**			Correlation $\in$ **[0.65,0.75)**			Correlation $\in$ **[0.35,0.45)**		
	Beta(1,4)	Permutation		Beta(1,74)	Permutation		Beta(1,282)	Permutation
ENT/ANT(%)	**0.60%**	**0.49%**	ENT/ANT(%)	**11.10%**	**12.48%**	ENT/ANT(%)	**42.30%**	**49.06%**
ENT	4	3	**ENT**	72	81	ENT	275	319
ENT_CI.low	4	3	ENT_CI.low	72	67	ENT_CI.low	274	293
ENT_CI.up	4	3	ENT_CI.up	72	93	ENT_CI.up	276	343
MWSL	**0**.**012740**	**0**.**015879**	MWSL	**0**.**000693**	**0**.**000620**	MWSL	**0**.**000182**	**0**.**000155**
MWSL_CI.up	0.012797	0.017769	MWSL_CI.up	0.000696	0.000710	MWSL_CI.up	0.000183	0.000170
MWSL_CI.low	0.012684	0.014545	MWSL_CI.low	0.000690	0.000536	MWSL_CI.low	0.000181	0.000138

Correlation $\in$ **[0.85,0.95)**			Correlation $\in$ **[0.55,0.65)**			Correlation $\in$ **[0.25,0.35)**		
	Beta(1,14)	Permutation		Beta(1,126)	Permutation		Beta(1,416)	Permutation
ENT/ANT(%)	**2.10%**	**1.73%**	ENT/ANT(%)	**18.90%**	**20.68%**	ENT/ANT(%)	**62.37%**	**66.60%**
ENT	14	11	ENT	123	134	ENT	405	433
ENT_CI.low	14	10	ENT_CI.low	122	125	ENT_CI.low	404	440
ENT_CI.up	14	13	ENT_CI.up	123	143	ENT_CI.up	407	433
MWSL	**0**.**003657**	**0**.**004482**	MWSL	**0**.**000407**	**0**.**000373**	MWSL	**0**.**000123**	**0**.**000108**
MWSL_CI.up	0.003673	0.004964	MWSL_CI.up	0.000409	0.000410	MWSL_CI.up	0.000124	0.000117
MWSL_CI.low	0.003641	0.003900	MWSL_CI.low	0.000405	0.000328	MWSL_CI.low	0.000123	0.000097

Correlation $\in$ **[0.75,0.85)**			Correlation $\in$ **[0.45,0.55)**			Correlation $\in$ **(0,0.25)**		
	Beta(1,40)	Permutation		Beta(1,195)	Permutation		Beta(1,535)	Permutation
ENT/ANT(%)	**6.00%**	**5.49%**	ENT/ANT(%)	**29.24%**	**32.29%**	ENT/ANT(%)	**80.23%**	**85.26%**
ENT	39	36	ENT	190	210	ENT	522	554
ENT_CI.low	39	32	ENT_CI.low	189	192	ENT_CI.low	519	526
ENT_CI.up	39	41	ENT_CI.up	191	228	ENT_CI.up	524	586
MWSL	**0**.**001282**	**0**.**001596**	MWSL	**0**.**000263**	**0**.**000221**	MWSL	**0**.**000096**	**0**.**000088**
MWSL_CI.up	0.001276	0.001796	MWSL_CI.up	0.000264	0.000241	MWSL_CI.up	0.000096	0.000096
MWSL_CI.low	0.001287	0.001386	MWSL_CI.low	0.000262	0.000201	MWSL_CI.low	0.000095	0.000078

**Table 7:** BINNED data: ENT estimates with 95% confidence intervals in brackets, and type I error estimation from the permutation procedure for various simulated outcome measures: continuous, discrete-binary, discrete-count, time-to-event survival

Outcome type	Continuous			Binary			Count			Survival		
MESA Binned data	ENT	R (%)	Type I error (%)	ENT	R (%)	Type I error (%)	ENT	R (%)	Type I error (%)	ENT	R (%)	Type I error (%)
Identity	482 (442;506)	74	5.16	409 (379;443)	62	5.11	221 (198;256)	34	4.71	466 (431;505)	71	4.97
Multivariate log-normal	351 (316;388)	54	5.02	338 (310;380)	52	5.01	344 (305;377)	53	4.71	344 (309;366)	53	4.92
Multivariate normal	376 (345;423)	57	5.04	355 (318;395)	54	5.21	366 (326;404)	56	4.69	361 (331;397)	55	4.81

$$K=5000$$ permutations. ANT=655

**Table 8:** PCA on simulated data (ANT=655, *n*_*t*_ = 1,500, PCs = 350): ENT estimates with 95% confidence intervals in brackets, and type I error estimation from the permutation procedure for various simulated outcome measures: continuous, discrete-binary, discrete-count, time-to-event survival

Outcome type	Continuous			Binary			Count			Survival		
PCA simulated data	ENT	R (%)	Type I error (%)	ENT	R (%)	Type I error (%)	ENT	R (%)	Type I error (%)	ENT	R (%)	Type I error (%)
Identity	292 (271;314)	45	5.10	333 (307;360)	51	4.93	500 (469;542)	76	5.05	394 (371;427)	60	4.95
Multivariate log-normal	391 (365;417)	60	4.99	377 (347;401)	58	5.09	389 (360;414)	59	4.98	379 (356;410)	58	4.93
Multivariate normal	368 (340;401)	56	4.98	356 (332;384)	54	5.01	370 (345;398)	56	5.11	343 (320;368)	52	5.19

$$K=5000$$ permutations

**Fig. 1:**
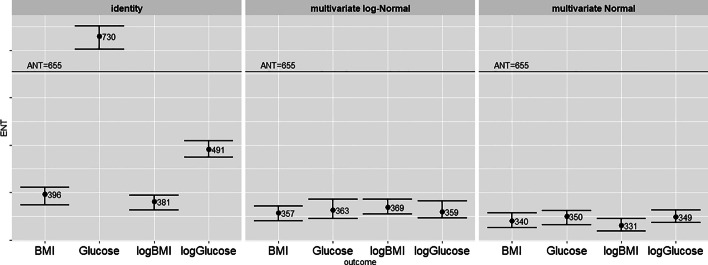
BINNED data: ENT across clinical outcome measures and for different approximations of the variates: original data, multivariate Normal, multivariate log-Normal. Error bars represent 95% confidence limits. *K*=10,000 permutations

**Fig. 2:**
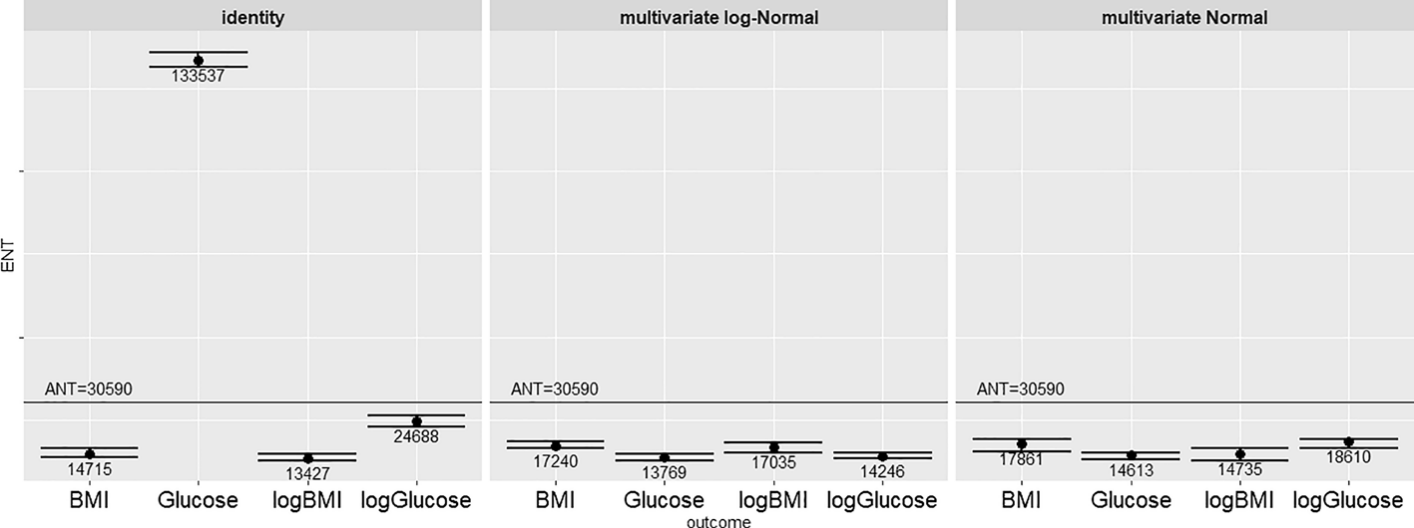
CPMG data: ENT across clinical outcome measures and for different approximations of the variates: original data, multivariate Normal, multivariate log-Normal. Error bars represent 95% confidence limits. *K*=10,000 permutations

**Fig. 3:**
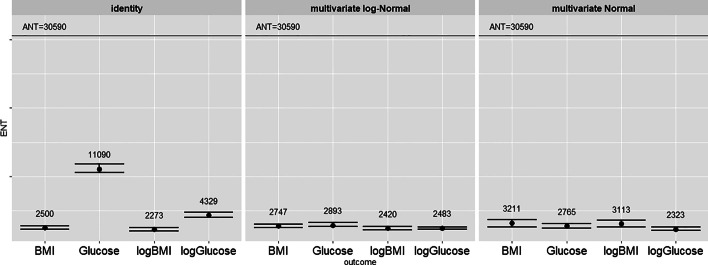
NOESY data: ENT across clinical outcome measures and for different approximations of the variates: original data, multivariate Normal, multivariate log-Normal. Error bars represent 95% confidence limits. *K*=10,000 permutations

**Fig. 4:**
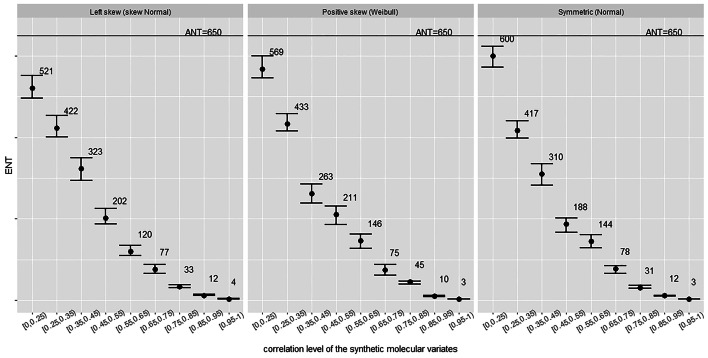
ENT for uncorrelated outcomes across correlated variates. Error bars represent 95% confidence limits. *K*=5,000 permutations

**Fig. 5:**
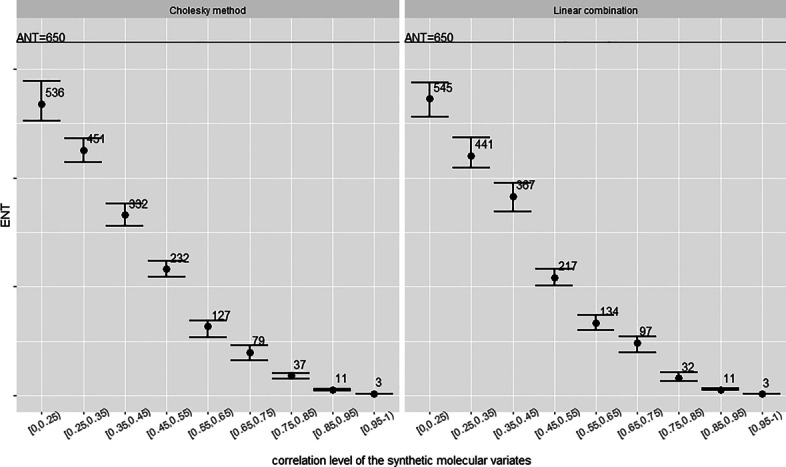
ENT for correlated outcome across correlated variates. Error bars represent 95% confidence limits. *K*=5,000 permutations

From the conventional permutation procedure applied to the BINNED data shown in Fig. 1, when the real features are considered, there is instability in the estimation of the ENT across the different outcomes, and in particular the ENT estimate for glucose is above the ANT. When the data are simulated from a multivariate log-Normal or Normal as described in Section “Parametric simulation methods” the ENT estimates are stable across the different outcomes and remain bounded below the total number of features with an average ENT around 350 and an R ratio around 50%. To assess the validity of this result in terms of redundancy of the set of features we considered principal component analysis (PCA) as an alternative method for estimating the ENT [6–8]. The cumulative proportion of variance explained by the first 350 PCs is around 99%. This is consistent with the interpretation that there are effectively 350 uncorrelated features in the data.

Figure 2 reports the ENT estimates for CPMG data. Without any transformations applied, there is a very large variation across the ENT estimates for the different outcomes, and in particular a very high and meaningless estimate for glucose levels which goes beyond R = 400%. On the other hand, when the set of features is simulated from the multivariate Normal and from the multivariate log-Normal distribution the corresponding ENT estimate is below the total number of features, and stable across different outcomes with an average ENT of around 16,000 features and an R ratio around 50%. In this case the usefulness of the proposed permutation method to estimate the ENT is clear since the PCA-based ENT estimate would be constrained to the maximum number of PCs ($n=3866$ i.e. max no. PC is *n*-1).

Figure 3 reports the ENT estimates for the NOESY data which are below R=100% but vary across outcomes when the original set of features is considered. When simulated features from the multivariate (log-) Normal distributions are considered we obtain lower ENT values than the ones from the CPMG data, with an average ENT of around 2700 features and an R ratio around 9%. This result was expected due to the reduced influence of broad signals in CPMG spectra compared to NOESY, which is linked to a weaker covariance structure. By applying a PCA to the NOESY data the cumulative proportion of variance explained by the first 2,700 PCs is around 99%, and this is in line with our findings.

Next, by exploiting the approximation method described in Section “Practical approximation of the ENT”, we derive the proposed $\text {Meff}_{\text {MWSL}}$. Table 3 provides this estimate compared to the available alternative methods proposed by [7–9], and [10], and the ENT estimate from the permutation procedure which is the averaged estimate of the results obtained via the multivariate and log-multivariate Normal transformations. Considering the complexity of the eigenvalue structure in cases of very large data sets, the proposed $\text {Meff}_{\text {MWSL}}$ in most cases seems to be able to consistently quantify, at least approximately, the correlation structure of the metabolomic variates. Based on this Meff estimate, to derive the MWSL and its confidence limits we simulate from a Beta(1,$\text {Meff}_{\text {MWSL}}$) which lets us obtain MWSL estimate of the same order of magnitude as those from the permutation procedure as shown in Table 4.

### Simulation study

We now broaden the investigation by considering various correlation levels of the set of molecular variates as well as cases of correlation between the outcome and the variates. At first we generate various sets of variates, each of these with a specific and well bounded correlation level. This is performed following the algorithm described in Section “Parametric algorithm to generate synthetic variates”. Specifically, we generated nine sets of variates covering the whole range of positive correlation levels. Next, we generate outcomes both correlated and uncorrelated to the variates which we will employ within the permutation procedure to estimate the ENT across the various sets of correlated molecular variates. *Uncorrelated outcomes* of different shapes are easily simulated via parametric distributions such as the Normal distribution for a symmetric outcome, the Skew-Normal distribution for a left skewed outcome, and a Weibull distribution for a right skewed outcome. Figure 4 shows the ENT estimates in the case of correlated variates and uncorrelated outcomes. Simulated *correlated outcomes* can be obtained as a linear combination of a few randomly chosen molecular variates with added noise, or via procedures based on Cholesky decomposition as is performed when simulating correlated features following the algorithm detailed in Section “Parametric algorithm to generate synthetic variates”. Figure 5 show the ENT estimates from the permutation procedure for the various sets of synthetic molecular variates and the correlated simulated outcomes. We conclude that correlation to the outcome makes no discernible difference to relationships between ENT and variate-variate correlation. Lastly, we apply the $\text {Meff}_{\text {MWSL}}$ approximation to derive the results in Tables 5 and 6. The ENT from the permutation procedure is averaged from the results in Figs. 4 and  5. In this simulated environment, the $\text {Meff}_{\text {MWSL}}$ approximation outperforms other available methods and describes well the permutation-based ENT estimates.

## Validation of the approach

A type I error (false-positive) occurs when a true null hypothesis is rejected. To check whether the permutation procedure accounts for the FWER at the $\alpha$ level, for each metabolic variate and across the permutation replicates, we measure the type I error rate as the number of occurrences having a *p* value less or equal to the MWSL. Rather than the original real-data outcomes we test the multivariate (log-)Normal permutation procedure to calculate the MWSL using various synthetic outcomes. In particular, we employ a continuous outcome from a Normal distribution, a discrete-binary outcome from a Binomial distribution, a discrete-count outcome from a Poisson distribution, and a time-to-event survival outcome from the Cox proportional hazards model as in [21]. We benchmark our result on the MESA BINNED data but also on a set of synthetic variates obtained via a nonparametric approach using PCA (see Section 6.2). We divide the data into test and non-test sets, compute a PCA model of the non-test data, and predict the test data based on this model. This approach allows us to generate synthetic data based on the structure of the real data without involving bootstrap/permutation methods [22] which we already employ to estimate the MWSL. Following the algorithm of Section 7.2 applied to the MESA BINNED data, we define the *test* and the *nontest* set by randomly sampling $n_t=1,500$ and $n_{{\bar{t}}}=3500-1500=2,000$ observations, respectively. From the PCA on the *nontest* set we select 350 PCs to be used to build the simulated *test* set of molecular variates ${{\hat{X}}}_t$. Table 7 and Table 8 confirm that the MWSL procedure effectively controls the FWER close to the (default) $\alpha$-level of 5%.

## Conclusions

In this paper we focus on assessing univariate test significance in multi collinear omics data by estimating a significance level threshold controlling the family wise error rate. The proposed procedure is based on an iterative permutation approach via univariate regression models while other measures of association may be used when appropriate. The molecular variates are simulated via parametric methods such as multivariate Normal and multivariate log-Normal distributions to retain the correlation structure in the data, while controlling the false positive rate at the desired level. When the permutation procedure is applied to the approximated data the MWSL is stable across outcome measures with diverse properties.

In MWAS, the metabolic profiles often exhibit a high degree of collinearity, and this is supported by our finding that in all scenarios considered, when parametric methods are applied to approximate the structure of the data, the MWSL estimated through the permutation procedure is larger than the threshold obtained via a metabolome-wide Bonferroni or Sidak corrections. Therefore, the corresponding ENT is always less than the actual number of tests as it mainly depends on the extent of correlation within the data. The extent of collinearity is summarized by the *R* ratio (%) of effective to actual number of tests. For the examples in this paper, *R* was found to be around 50% for the CPMG data (high-resolution and BINNED version), and around 9% for the NOESY high resolution. This is consistent with the expected higher degree of correlations between spectral variables in the NOESY data. As with other approaches, the proposed closed-form Meff approximation to the permutation-based ENT could be tentatively interpreted as the number of independent metabolic processes exhibited by the system. Both the MWSL or the Meff estimate can be employed downstream of the analysis to identify differentially regulated metabolites.

## Supplementary Information

**Additional file 1**. R tutorial MWSL.

## Data Availability

MWSL is an open-source R software package avaiable at https://github.com/AlinaPeluso/PhenoMeNal. Within the package we made available the lower resolution CPMG data referred to in the text as MESA BINNED data. An R tutorial is available as a supplementary material.

## Appendix

### Parametric algorithm to generate synthetic variates

- Step (1): Generate a square $(M \times M)$ correlation matrix *A* assuming all variables have unit variance, i.e. the *M* elements on the diagonal are 1s. The $[M(M-1)]/2$ elements of the upper triangular matrix are sampled from Uniform distributions bounded by a certain interval, e.g. high correlation level within the interval [0.75,0.85], medium correlation in [0.45,0.55], or low correlations in [0.25,0.35]. The lower triangle elements are copied from the upper triangle.
- Step (2): As the $\lambda$s of *A* are required to be greater than zero, compute *S* as the nearest positive definite to the correlation matrix *A* achieving $\{\min {{\left\| A-S\right\| }_F: \, S \, \text {is a correlation matrix}}\}$, where ${\left\| A\right\| }^2_F =\sum _{i,j} a^2_{ij}$ as described by [23].
- Step (3): Derive the lower triangular matrix *L* via Cholesky decomposition of matrix *S* such that *S* = *LL*’.
- Step (4): *M* multivariate Normal features with zero means result from the product *ZL* between the $(n \times M)$ matrix *Z* of *M* random N(0,1) i.i.d. features, and the $(M \times M)$ lower triangular matrix *L*. The correlations of the simulated features are very close to those assigned in matrix *A*.

### Nonparametric algorithm to generate synthetic variates

- Step (1): By randomly sampling $n_t$ observations from the original data matrix of variates *X*, construct the $(n_t\times M)$ *test* set of variates $X_t$, and the $(n_{{\bar{t}}} \times M)$ *nontest* set $X_{{\bar{t}}}$, with $n_t<n$ and $n_{{\bar{t}}}=n-n_t$.
- Step (2): Standardise the *test* and the *nontest* sets by subtracting their respective vectors of column means i.e. $\mu _t$ and $\mu _{{\bar{t}}}$, and dividing by their standard deviations i.e. $\sigma _t$ and $\sigma _{{\bar{t}}}$, to respectively obtain $Z_t$ and $Z_{{\bar{t}}}$.
- Step (3): Compute PCA over the *nontest* set by applying singular value decomposition (SVD) such that $Z_{{\bar{t}}}=U_{{\bar{t}}} \Sigma _{{\bar{t}}} V_{{\bar{t}}}^T$, where $V_{{\bar{t}}}^T$ is the $(M \times M)$ matrix of loadings, while the PC scores are obtained as the product between the $(n_{{\bar{t}}} \times n_{{\bar{t}}})$ matrix $U_{{\bar{t}}}$ of eigenvectors of $Z_{{\bar{t}}} Z_{{\bar{t}}}^T$, and the $(n_{{\bar{t}}} \times M)$ diagonal matrix $\Sigma _{{\bar{t}}}$.
- Step (4): Use the *nontest* loadings $V_{{\bar{t}}}$ combined with the test $X_t$ to compute the $(n_t \times M)$ matrix ${{\hat{U}}}_t {\hat{\Sigma }}_t$ of PC predicted scores for the *test* set, i.e. ${{\hat{U}}}_t {\hat{\Sigma }}_t = X_t V_{{\bar{t}}}$.
- Step (5): Build the $(n_t \times M)$ simulated *test* set of variates $\hat{Z_t}$ as the product of the predicted scores from Step (4), and the matrix of loadings $V_{{\bar{t}}}^T$ from Step (3) such that ${{\hat{Z}}}_t= {{\hat{U}}}_t {\hat{\Sigma }}_t V_{{\bar{t}}}^T$. We note that *S* PCs, with $S \le M$, can be selected to be used in the predictions, thus ${{\hat{Z}}}_t$ would result from the product of the $(n_t \times S)$ matrix of PCs and the $(S \times M)$ matrix of loadings.
- Step (6): From the simulated *test* set of standardised features ${{\hat{Z}}}_t$ compute the $(M \times M)$ set of simulated features as ${{\hat{X}}}_t = {{\hat{Z}}}_t \sigma _t + \mu _t$.

To simulate the set of variates in such way the sample size of the data should be large enough for the data to be split between the test and the nontest set, and no missing values are allowed. Nevertheless, a possible extension of this method would consider the Nonlinear Iterative Partial Least Squares (NIPALS) algorithm as a modified PCA to accommodate missing values [24].

## Abbreviations

MWAS: Metabolome-Wide Association studies
FDR: false discovery rate
FWER: family-wise error rate
MWSL: metabolome-wide significance level
ENT: effective number of test
ANT: actual number of test

## Footnotes

1 Binning is a common data reduction approach in NMR metabolomics.
