# Supplementary material for: Multiple-testing correction in metabolome-wide association studies
Source: BMC Bioinformatics. 2021 Feb 12;22:67. doi: 10.1186/s12859-021-03975-2 (PMC7881646; doi:10.1186/s12859-021-03975-2)
Supplement: Supplementary file 1 — Additional file 1. R tutorial MWSL. [file 12859_2021_3975_MOESM1_ESM.pdf]

PhenoMeNal (Phenome and Metabolome aNalysis) - processing and analysis of molecular phenotype data generated by metabolomics applications

Permutation-based and closed-form-expression tools for the estimation of metabolome-wide significance level (MWSL) and corresponding effective number of tests for (correlated) metabolomics data.

Section 0: Import the R package

The `MWSL` R package allows for

- Estimation of permutation-based MWSL and corresponding effective number of tests (ENT)
- Estimation of closed-form-expression MWSL and corresponding effective number of tests (Meff)
- Identification of differentially regulated metabolomics variates for a specific clinical outcome

```
devtools::install_github("AlinaPeluso/PhenoMeNal", subdir="MWSL")
library(MWSL)
```

Section 1: Exploratory analysis

We aim to investigate the association between human serum 1H NMR metabolic profiles and various clinical outcomes in the Multi-Ethnic Study of Atherosclerosis (MESA).

The reference paper considers three sets of NMR spectra data: (1) a standard water-suppressed one-dimensional spectrum (NOESY), and (2) a Carr-Purcell-Meiboom-Gill spectrum (CPMG), and (3) a lower resolution version of the CPMG data (BINNED). The BINNED version consists of `M = 655` features, while the NOESY and CPMG contain `M = 30,590` features. The BINNED data sample comprises of `n = 3500` individuals, while the NOESY and CPMG data have `n = 3,867` individuals.

To illustrate the package capabilities, here we reproduce the results for the BINNED version of the data.

```
data("MESA_binned")
```

Metabolomics variates

For this specific analysis the metabolomics variates are anonimised and simply referred to as `V1,V2,...,V655`.

```
features <- MESA_binned[,23:(ncol(MESA_binned)-1)]
```

Descriptive statistics of the metabolomics variates:

```
t(round(sapply(features, function(x) c(mean=mean(x),sd=sd(x),median=median(x),min=min(x),max=max(x)
)),2))
```

feature	mean	sd	median	min	max
V1	0.13	0.98	0.03	-2.44	5.33
V2	0.15	0.78	0.05	-1.66	3.73
V3	0.05	0.95	0.02	-3.17	3.83
V4	2.85	0.99	2.79	0.04	6.38
V5	1.75	0.99	1.73	-2.29	5.85
…	…	…	…	…	…
V651	0.04	1.01	0.03	-7.21	4.25
V652	0.01	1.01	0.04	-6.48	4.63
V653	0	1.01	0.03	-4.81	5.51
V654	0.03	1.01	0.03	-3.74	4.08
V655	0.02	1.01	0.03	-4.09	4.54

Fixed effects confounders

Briefly, the cohort includes participants (51\% females, 49\% males), aged 44-84 years, (mean age of 63 years) from four different ethnic groups: Chinese-American, African-American, Hispanic, and Caucasian, all recruited between 2000-2002 at clinical centres in the United States and free of symptomatic cardiovascular disease at baseline. Demographic, medical history, anthropometric, and lifestyle data, as well as serum samples were collected, together with information on diabetes, and lipid and blood pressure treatment.

```
MESA_binned$male <- ifelse(MESA_binned$sex<2,1,0)
confounders <- MESA_binned[,c("age","male","height","ethnicityH","ethnicityAA","ethnicityCA","smokingF","smokingC","ldl_chol","hdl_chol","sbp","bp_treatment","diabetes","lipids_treatment")]
```

Descriptive statistics of the clinical outcomes measures:

```
t(round(sapply(MESA_binned[,c(7,9:22,ncol(MESA_binned))], function(x) c(mean=mean(x),sd=sd(x),median=median(x),min=min(x),max=max(x))),3))
```

confounder	mean	sd	median	min	max
age	62.888	10.327	64	44	84
height	166.433	10.237	166.3	123.8	196.7
ethnicityC	0.385	0.487	0	0	1
ethnicityH	0.234	0.424	0	0	1
ethnicityAA	0.255	0.436	0	0	1
ethnicityCA	0.127	0.333	0	0	1
smokingN	0.503	0.5	1	0	1
smokingF	0.121	0.326	0	0	1
smokingC	0.376	0.485	0	0	1
ldl_chol	117.678	31.043	116	20	315
hdl_chol	51.3	14.42	49	21	133
sbp	126.922	21.54	124	77	218
bp_treatment	0.376	0.484	0	0	1
diabetes	0.135	0.342	0	0	1
lipids_treatment	0.167	0.373	0	0	1
male	0.489	0.5	0	0	1

Clinical outcomes measures

The outcomes of interest are glucose concentrations and the body mass index or BMI.

```
glucose <- MESA_binned[,1]; log_glucose <- MESA_binned[,2]; bmi <- MESA_binned[,7]; log_bmi <- MESA
_binned[,8];
outcomes <- MESA_binned[,c(1:4)]
```

Descriptive statistics of the clinical outcomes measures:

```
t(round(sapply(MESA_binned[,c(1:4)], function(x) c(mean=mean(x),sd=sd(x),median=median(x),min=min(x
),max=max(x))),2))
```

outcome	mean	sd	median	min	max
glucose	97.55	29.6	90	38	507
logGlucose	4.55	0.22	4.5	3.64	6.23
BMI	28.14	5.39	27.34	15.36	61.86
logBMI	3.32	0.18	3.31	2.73	4.12

Plot of the distributions of the clinical outcomes measures:

```
par(mfrow=c(2,4))
for (i in 1:ncol(outcomes)){hist(outcomes[,i],main=names(outcomes)[i],xlab=NULL)}
for (i in 1:ncol(outcomes)){boxplot(outcomes[,i],main=names(outcomes)[i],xlab=NULL)}
```

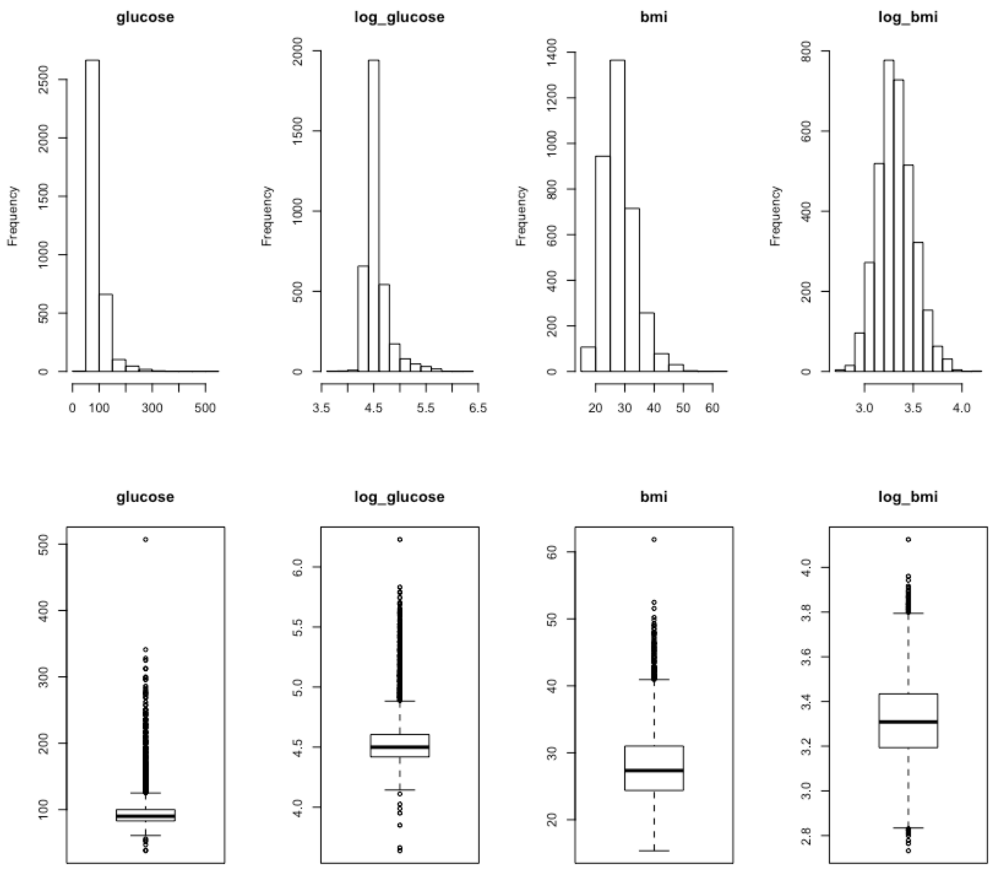

Section 2: Permutation-based MWSL and ENT estimation

`MWSL::FWERperm` performs the estimation of permutation-based metabolome-wide significance level (`MWSL`) and the corresponding effective number of tests (`ENT`). The procedure controls the FWER at the α level. The type I error rate (false-positive rate) is measured as the number of occurrences of having a p-value less or equal than the MWSL, that is when a true null hypothesis is being rejected.

Arguments:

- `outcome` a vector of `n` data point values of a continuous (both symmetric and skewed), discrete binary (0/1) or count outcome, or a data frame with `n` observations and column variables `time` (or `time1` and `time2`) and `status` for a time-to-event survival outcome.
- `features` a data frame of `n` observations (rows) and `M` features e.g. metabolic profiles (columns).
- `confounders` an optional data frame of `n` observations (rows) and `P` fixed effects confounders (columns). Default to `confounders` = `NULL` .
- `methods` an optional string which can take values `'identity'` if no transformation is applied to the data, or `'mN'` (or `'mlogN'`) when the set of features is simulated via a multivariate Normal (or multivariate log-Normal) distribution.

Default to `methods` = `'mN'` .

- `n.permutation` an optional numeric value. Default to `n.permutation` =10,000.
- `alpha` an optional probability value. Default to `alpha` =0.05.
- `verbose` an optional logical value which allows output some status messages while computing. Default to `verbose` = `TRUE` .

Outputs:

- `matPvals` the matrix of p-values for the `M` features (columns) and the `n.permutation` (rows).
- `q` the vector of minimum p-values of length `n.permutation` .
- `res` the vector of result estimates:
    - `MWSL` = metabolome-wide significance level (MWSL);
    - `MWSL_CI.up` = upper value `alpha` \%-confidence interval MWSL;
    - `MWSL_CI.low` = lower value `alpha` \%-confidence interval MWSL.
    - `ENT` = effective number of tests (ENT);
    - `ENT_CI.up` = upper value `alpha` \%-confidence interval ENT;
    - `ENT_CI.low` = lower value `alpha` \%-confidence interval ENT;
    - `R.percent` = ENT/M.
- `t1err.percent` the estimated type I error (\%).

Optimal performances in terms of computational time are achieved when the procedure runs on a multi-core computer as parallel computing is applied within the function to deal with the heaviest steps.

Run the function across the clinical outcomes measures:

```
methods <- c('identity','mN','mlogN')
mat <- matrix(NA,3,8)
colnames(mat) <- c('MWSL','MWSL_CI.up','MWSL_CI.low','ENT','ENT_CI.up','ENT_CI.low','R.percent','t1
err.percent')
rownames(mat) <- methods
rmesa_FWERperm <- list(glucose=mat,log_glucose=mat,bmi=mat,log_bmi=mat)

rmesa_pval <- list(glucose=mat,log_glucose=mat,bmi=mat,log_bmi=mat)
rmesa_FWERperm <- list(glucose=mat,log_glucose=mat,bmi=mat,log_bmi=mat)
allres_mesa <- list()
for (j in 1:length(methods)){
  for (i in 1:ncol(outcomes)){
    rmesa <- FWERperm(outcome=outcomes[,i],
                      features=features,
                      confounders=confounders,
                      n.permutation=60,
                      method=methods[j],
                      verbose=F)
    allres_mesa[[3*(i-1)+j]] <- rmesa
    rmesa_FWERperm[[i]][j,1:7] <- rmesa$res
    rmesa_FWERperm[[i]][j,8] <- rmesa$t1err.percent
  }
}
df.names <- expand.grid(methods, names(outcomes))
names(allres_mesa) <- paste(df.names$Var1, df.names$Var2,sep='.')
```

Explore the results:

```
rmesa_FWERperm
```

glucose

	MWSL	MWSL_CI.up	MWSL_CI.low	ENT	ENT_CI.up	ENT_CI.low	R.percent	t1err.percent
identity	0.0000685	0.0000665	0.0000713	729.803	751.496	701.647	111.42	4.95
mN	0.0001430	0.0001380	0.0001508	349.737	362.19	331.658	53.39	4.97
mlogN	0.0001376	0.0001296	0.0001447	363.358	385.699	345.604	55.47	5.14

logGlucose

	MWSL	MWSL_CI.up	MWSL_CI.low	ENT	ENT_CI.up	ENT_CI.low	R.percent	t1err.percent
identity	0.0001018	0.0000982	0.0001053	491.344	508.917	474.867	75.01	5.06
mN	0.0001434	0.0001378	0.0001483	348.567	362.855	337.154	53.22	5.07
mlogN	0.0001392	0.0001310	0.0001442	359.09	381.818	346.816	54.82	4.98

BMI

	MWSL	MWSL_CI.up	MWSL_CI.low	ENT	ENT_CI.up	ENT_CI.low	R.percent	t1err.percent
identity	0.0001262	0.0001216	0.0001335	396.188	411.0372	374.544	60.49	5.18
mN	0.0001470	0.0001401	0.0001534	340.142	357.0032	325.845	51.93	5.20
mlogN	0.0001402	0.0001345	0.0001468	356.707	371.7413	340.712	54.46	5.00

logBMI

	MWSL	MWSL_CI.up	MWSL_CI.low	ENT	ENT_CI.up	ENT_CI.low	R.percent	t1err.percent
identity	0.0001313	0.0001266	0.0001377	380.688	394.8619	363.169	58.12	5.08
mN	0.0001512	0.0001447	0.0001567	330.715	345.6358	319.064	50.49	5.12
mlogN	0.0001356	0.0001296	0.0001410	368.722	385.7733	354.6	56.29	4.92

The permutation procedure provides strong control of the FWER at the α level set it to 5% while it also incorporates the joint dependence structure between the test statistics.

We can inspect the distribution of p-values corresponding to the case where the multivariate Normal distribution is employed to simulate the set of features

```
hist(allres_mesa[["mN.glucose"]][["matPvals"]],main="Plot p-values under the null",breaks=50,xlab=N
ULL)
```

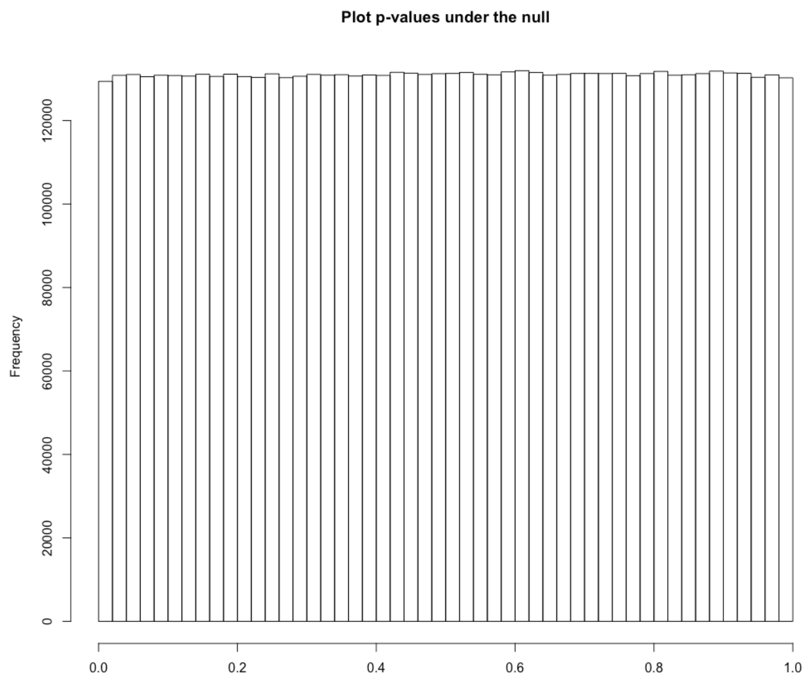

The p-values have a uniform distribution as we have re-sampled under the true null hypothesis of no association.

We can also inspect the distribution of the minimum p-values corresponding to the case where the multivariate Normal distribution is employed to simulate the set of features

```
hist(allres_mesa[["mN.glucose"]][["q"]],main="Plot minimum p-values",breaks=150,xlab=NULL)
op <- par(cex = 1.5); alpha=0.05
abline(v=rmesa_FWERperm$glucose[1,1],col="red",lwd=5)
abline(v=rmesa_FWERperm$glucose[2,1],col="blue",lwd=5)
abline(v=rmesa_FWERperm$glucose[3,1],col="brown",lwd=5)
abline(v=1-(1-alpha)^(1/ncol(features)),col="green",lwd=5)
abline(v=alpha/ncol(features),col="orange",lwd=5)
legend("topright",c('perm_id','perm_mN','perm_mlogN','Sidak','Bonferroni'),fill=c("red","blue","bro
wn","green","orange"))
```

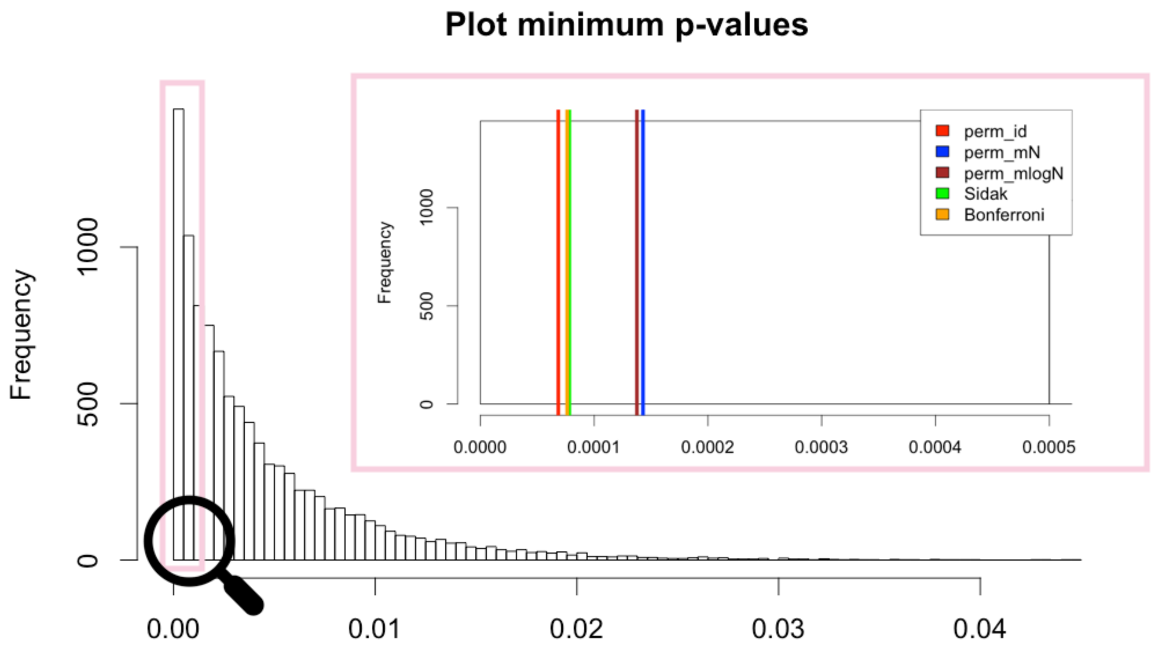

The procedure is less conservative than the Bonferroni or Sidak correction.

Plot of the ENT estimates from the permutation procedure:

```
df_rmesa_FWERperm <- do.call(rbind,rmesa_FWERperm)
df1_rmesa_FWERperm<- data.frame(
  outcome = c('glucose','glucose','glucose',
              'logGlucose','logGlucose','logGlucose',
              'BMI','BMI','BMI',
              'logBMI','logBMI','logBMI'),
  type = c('identity','multivariate Normal','multivariate log-Normal',
           'identity','multivariate Normal','multivariate log-Normal',
           'identity','multivariate Normal','multivariate log-Normal',
           'identity','multivariate Normal','multivariate log-Normal'),
  ENT = c(df_rmesa_FWERperm[,4]),
  ENT.ciUP = c(df_rmesa_FWERperm[,5]),
  ENT.ciLOW = c(df_rmesa_FWERperm[,6]))
(plot_res.MESA_co <- ggplot(data=df1_rmesa_FWERperm,aes(x=outcome,y=ENT)) +
    facet_grid(~ type) +
    geom_hline(yintercept=ncol(features)) +
    annotate("text",x='BMI',y=(ncol(features)+15),label='ANT=655') +
    geom_text(mapping=aes(label=round(ENT,0)),hjust=-.5)+
    geom_point(size=3) +
    geom_errorbar(aes(ymin=ENT.ciLOW,ymax=ENT.ciUP),size=1) +
    theme(text = element_text(size=20)) +
    theme(legend.position="bottom") +
    theme(axis.text.x = element_text(angle=30, hjust=1)) +
    theme(axis.text.y = element_blank()) +
    ggtitle("MESA_binned data - all clinical outcomes")
)
```

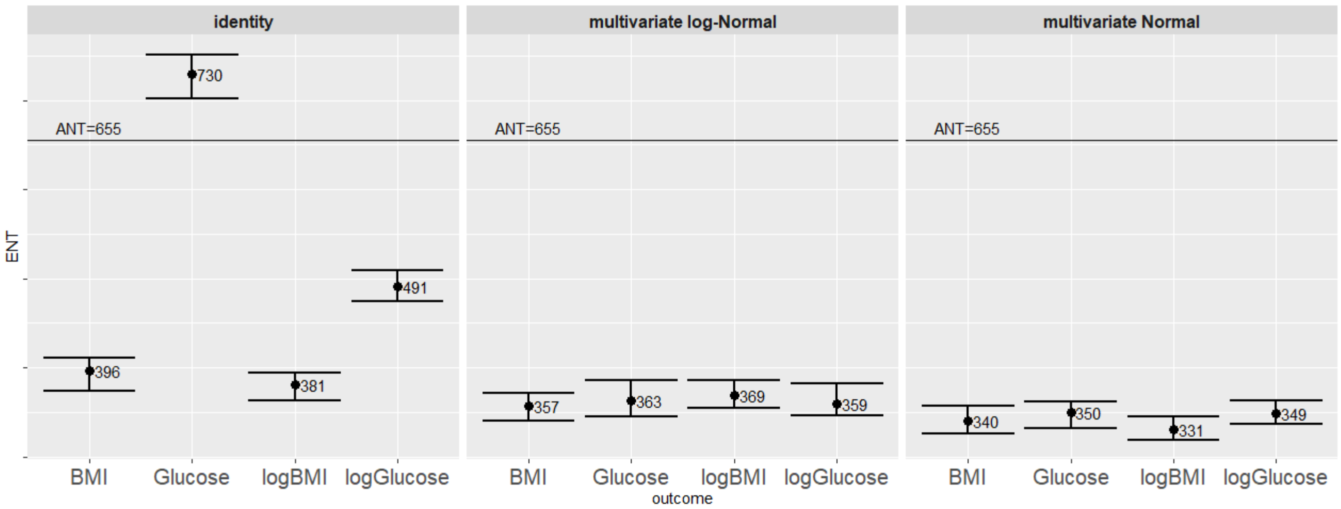

From the conventional permutation procedure applied to the BINNED data, when the real features are considered, there is instability in the estimation of the ENT across the different outcomes, and in particular the ENT estimate for glucose is above the ANT. When the feature data are simulated from a multivariate log-Normal or Normal distribution, the ENT estimates are stable across the different outcomes and remain bounded below the total number of features with an average ENT of 352 and an R ratio of 53.8%.

Section 3: Closed form expression eigenvalues-based MWSL and ENT estimation

The empirical method of computing the permutation test p-value is hampered by the fact that a very large number of permutation is required to correctly estimate small, and therefore interesting p-values. Thus, the `MWSL::Meff` allows for an efficient approximation alternative. To distinguish from the effective number of non-redundant variates from the permutation procedure which has been defined as `ENT`, here we refer to the estimate from this practical approximation approach as `Meff`. The approximation is based on the spectral decomposition of the correlation matrix of the metabolomics variates, and allows for the comparison of the proposed estimate with methods of interest from the genomics field which proposed a similar formulation based on the same concept.

Arguments:

- `features` a data frame of `n` observations (rows) and `M` features e.g. metabolic profiles (columns).
- `methods` a string with possible values `ecorr` (empirical correlation), or `scorr` (shrinkage correlation). Default to `ecorr`.
- `alpha` an optional probability value. Default to `alpha` =0.05.
- `big.mat` an optional logic value to be set to `TRUE` when dealing with a very large set of features. Default to `FALSE`.

Outputs:

- `Meff_Nyholt` closed-form expression of the effective number of tests based on Nyholt (2004)
- `Meff_Liji` closed-form expression of the effective number of tests based on Li & Ji (2005)
- `Meff_Gao` closed-form expression of the effective number of tests based on Gao (2008)
- `Meff_Galwey` closed-form expression of the effective number of tests based on Galwey (2009)
- `Meff_Bonferroni` closed-form expression of the effective number of tests based on Bonferroni (1963)
- `Meff_Sidak` closed-form expression of the effective number of tests based on Sidak (1967)
- `Meff_MWSL` the proposed closed-form expression of the effective number of tests

- `res.Meff_MWSL` the vector of result estimates:
  - `MWSL` = metabolome-wide significance level (MWSL);
  - `MWSL_CI.up` = upper value `alpha` \%-confidence interval MWSL;
  - `MWSL_CI.low` = lower value `alpha` \%-confidence interval MWSL.
  - `ENT` = effective number of tests (ENT);
  - `ENT_CI.up` = upper value `alpha` \%-confidence interval ENT;
  - `ENT_CI.low` = lower value `alpha` \%-confidence interval ENT;
  - `R.percent` = ENT/M.

Run the function:

```
rmesa_Meff_ecorr <- Meff(features=features,
                         n.permutation=100000,
                         method='ecorr',
                         big.mat=FALSE,
                         alpha=0.05)
```

Explore the results:

```
mat.rmesa_Meff_ecorr <- rbind(Meff_Nyholt=rmesa_Meff_ecorr$Meff_Nyholt,
                              Meff_Liji=rmesa_Meff_ecorr$Meff_Liji,
                              Meff_Gao=rmesa_Meff_ecorr$Meff_Gao,
                              Meff_Galwey=rmesa_Meff_ecorr$Meff_Galwey,
                              Meff_Bonferroni=rmesa_Meff_ecorr$Meff_Bonferroni,
                              Meff_Sidak=rmesa_Meff_ecorr$Meff_Sidak,
                              Meff_MWSL=rmesa_Meff_ecorr$Meff_MWSL);
mat.rmesa_Meff_ecorr
```

Method	Estimate	R(%)
Meff_Nyholt	611	93%
Meff_Liji	226	35%
Meff_Gao	435	66%
Meff_Galwey	201	31%
Meff_Bonferroni	655	100%
Meff_Sidak	639	98%
Meff_MWSL	345	53%
ENT	352	54%

The closed-form `Meff` value is a good approximation of the `ENT` estimate from the permutation procedure.

The `MWSL` estimate can be access as

```
rmesa_Meff_ecorr$res.Meff_MWSL
```

MWSL	MWSL_CI.up	MWSL_CI.low	ENT_MWSL	ENT_MWSL_CI.up	ENT_MWSL_CI.low	R.percent
0.000145102	0.000147141	0.000142823	344.5863	350.0843	339.8103	52.61%

Section 4: Identification of outcome-specific differentially regulated metabolites

This section focus on identification of differentially regulated metabolomics variates directly linked to a specified outcome (e.g. disease risk). The procedure supports various outcome type i.e. continuous outcomes both symmetric and skewed, binary (0/1) outcome, countable outcome taking discrete values 0,1,2,3,$...$, as well as a survival time-to-event outcome.

Outcome type	Model	R function
continuous (symmetric)	OLS regression	stats::lm
continuous (skewed)	Median quantile regression	quantreg::rq(tau=.5)
binary (0/1)	Logistic regression	stats::glm(family="binomial")
count (equidispersed)	GLM Poisson regression	stats::glm(family="poisson")
count (overdispersed)	Negative Binomial regression	glm(family="negative.binomial")
survival (time-to-event)	Cox proportional hazards regression	survival::coxph

Once the appropriate analysis has been performed the metabolites of interest can be identified comparing the respective p-value to the the adjusted thresold (MWSL or Meff). When the raw p-value corresponding to a certain feature is smaller than the adjusted thresold we identify that metabolomic variate as significant. For the other metabolites we conclude that there is no association between the changes in the outcome variable and the shifts in these features.

Arguments:

- `outcome` a vector of `n` data point values of a continuous (both symmetric and skewed), discrete binary (0/1) or count outcome, or a data frame with `n` observations and column variables `time` (or `time1` and `time2`) and `status` for a time-to-event survival outcome.
- `features` a data frame of `n` observations (rows) and `M` features e.g. metabolic profiles (columns).
- `confounders` an optional data frame of `n` observations (rows) and `P` fixed effects confounders (columns). Default to `confounders` = `NULL`.
- `MWSL` metabolome-wide significance level (MWSL) estimated via the permutation-based approach (see `MWSL::FWERperm`) or via the via the closed-form-expression approach (see `MWSL::Meff`)
- `alpha` an optional probability value, default=0.05
- `vennPlot` an optional logic value to be set to `TRUE` for the visualisation of the Venn-plot for the total count of differentally regulated metabolomics variates when employing the Bonferroni, the Benjamini-Hochberg (FDR), and the MWSL (FWER) correction, respectively
- `verbose` an optional logic value to suppress some output status messages. Default to `TRUE`.

Outputs:

- `res.DE_count` total count of differentally regulated metabolomics variates when employing the Bonferroni, the Benjamini-Hochberg (FDR), and the MWSL (FWER) correction, respectively.
- `res.DE_names` names of the of differentally regulated metabolomics variates when employing the Bonferroni, the

Benjamini-Hochberg (FDR), and the MWSL (FWER) correction, respectively.

- `Venn_plot Venn-plot` visualisation tool for the total count of differentally regulated metabolomics variates when employing the Bonferroni, the Benjamini-Hochberg (FDR), and the MWSL (FWER) correction, respectively.

Run the function for all the clinical outcomes measures:

```
rmesa_DEtest <- rmesa_DEtest_count  <- list()
names(rmesa_DEtest) <- c('glucose','logGlucose','BMI','logBMI')
for (i in 1:ncol(outcomes)){
  res_DEtest <- DEtest(outcome=outcomes[,i],
                      features=features[,1:10],
                      confounders=confounders,
                      MWSL=0.000145102,
                      alpha=0.05,
                      vennPlot=TRUE)
  rmesa_DEtest[[i]]<- res_DEtest
  rmesa_DEtest_count[[i]]<- res_DEtest$res.DE_count
}
```

Explore the results:

A Venn plot is generated for the visualisation of the number of identified differentially regulated metabolomics variates when employing the Bonferroni, the Benjamini-Hochberg (BH, FDR), and the MWSL (FWER) correction.

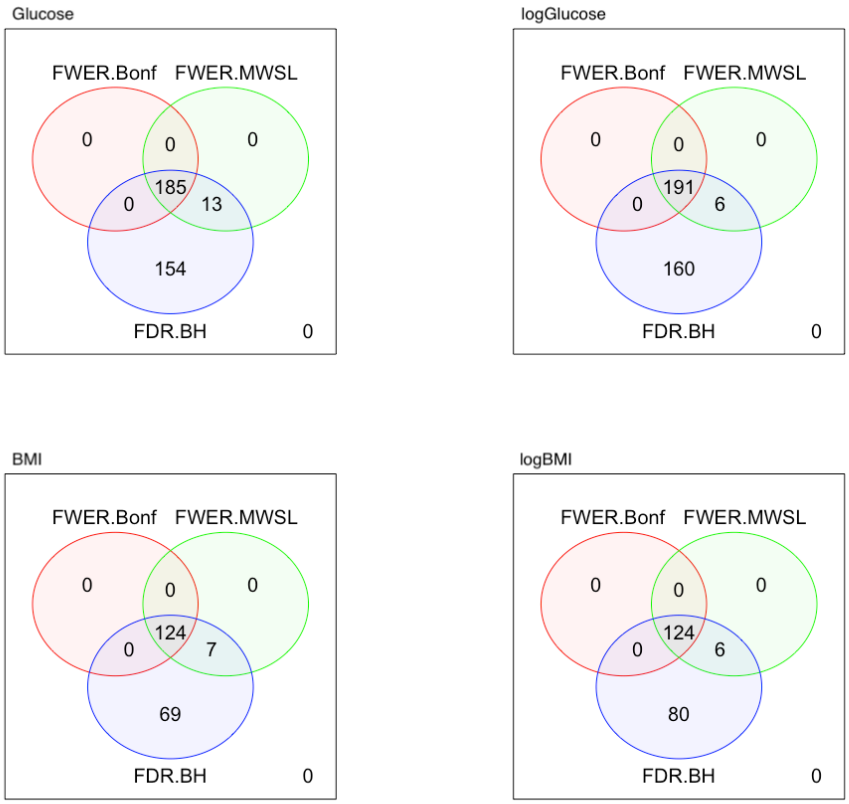

The counts of the differentially regulated metabolomics variates for each clinical outcomes can be accessed as

```
res.mesa_DEtest_count <- do.call(rbind, rmesa_DEtest_count)
rownames(res.mesa_DEtest_count) <- c('glucose','logGlucose','BMI','logBMI')
res.mesa_DEtest_count
```

	FWER.Bonf	FWER.MWSL	FDR.BH
glucose	185	198	352
logGlucose	191	197	357
BMI	124	131	200
logBMI	124	130	210

The FWER approach based on the MWSL estimation allows for the identification of a number of differentially regulated variates less than the too liberal BH correction and greater than the highly conservative Bonferroni correction. The number of differentially regulated variates is stable when considering transformations of the clinical outcome measures.

The list of differentially regulated metabolomics variates identified via the MWSL approach for a specific clinical outcome e.g. `glucose` can be accessed as

```
rmesa_DEtest[["glucose"]][["res.DE_names"]][["FWER.MWSL"]]
```

V1	V3	V4	V8	V9	...	V596	V597	V610	V618	V619
